# Malaria Infections Do Not Compromise Vaccine-Induced Immunity against Tuberculosis in Mice

**DOI:** 10.1371/journal.pone.0028164

**Published:** 2011-12-19

**Authors:** Marcela Parra, Steven C. Derrick, Amy Yang, JinHua Tian, Kristopher Kolibab, Miranda Oakley, Liyanage P. Perera, William R. Jacobs, Sanjai Kumar, Sheldon L. Morris

**Affiliations:** 1 Office of Vaccines Research and Review, Center for Biologics Evaluation and Review, USFDA, Bethesda, Maryland, United States of America; 2 Metabolism Branch, Center for Cancer Research, National Cancer Institute, National Institutes of Health, Bethesda, Maryland, United States of America; 3 Howard Hughes Medical Institute and Department of Microbiology and Immunology, Albert Einstein College of Medicine, Bronx, New York, United States of America; 4 Office of Blood Research and Review, Center for Biologics Research and Review, USFDA, Bethesda, Maryland, United States of America; Tulane University, United States of America

## Abstract

**Background:**

Given the considerable geographic overlap in the endemic regions for malaria and tuberculosis, it is probable that co-infections with *Mycobacterium tuberculosis* and *Plasmodium* species are prevalent. Thus, it is quite likely that both malaria and TB vaccines may be used in the same populations in endemic areas. While novel vaccines are currently being developed and tested individually against each of these pathogens, the efficacy of these vaccines has not been evaluated in co-infection models. To further assess the effectiveness of these new immunization strategies, we investigated whether co-infection with malaria would impact the anti-tuberculosis protection induced by four different types of TB vaccines in a mouse model of pulmonary tuberculosis.

**Principal Findings:**

Here we show that the anti-tuberculosis protective immunity induced by four different tuberculosis vaccines was not impacted by a concurrent infection with *Plasmodium yoelii* NL, a nonlethal form of murine malaria. After an aerogenic challenge with virulent *M. tuberculosis*, the lung bacterial burdens of vaccinated animals were not statistically different in malaria infected and malaria naïve mice. Multi-parameter flow cytometric analysis showed that the frequency and the median fluorescence intensities (MFI) for specific multifunctional T (MFT) cells expressing IFN-γ, TNF-α, and/or IL-2 were suppressed by the presence of malaria parasites at 2 weeks following the malaria infection but was not affected after parasite clearance at 7 and 10 weeks post-challenge with *P. yoelii* NL.

**Conclusions:**

Our data indicate that the effectiveness of novel TB vaccines in protecting against tuberculosis was unaffected by a primary malaria co-infection in a mouse model of pulmonary tuberculosis. While the activities of specific MFT cell subsets were reduced at elevated levels of malaria parasitemia, the T cell suppression was short-lived. Our findings have important relevance in developing strategies for the deployment of new TB vaccines in malaria endemic areas.

## Introduction

*Plasmodium falciparum* and *Mycobacterium tuberculosis* are among the world's most important tropical diseases. Malaria and tuberculosis are major global causes of morbidity and mortality with each causing 1–2 million deaths annually. The World Health Organization has reported that there are 300–500 million new cases of malaria and 9 million new cases of tuberculosis each year [1], [2]. Moreover, it has been estimated that one-third of the world's population is infected with latent TB. Given the substantial geographic overlap of endemic regions for these diseases and especially the large number of individuals with latent TB living in malaria-endemic regions, it is highly probable that co-infections with *M. tuberculosis* and *Plasmodium* species are common [3], [4]. This presumed high rate of malaria-TB co-infections could be problematic for the development of TB vaccines targeted for malaria-endemic areas of the world. Malaria parasites are known to be immunosuppressive and acute malaria infections have already been associated with decreased immune responses to meningococcal, Hib conjugate, and *Salmonella typhi* vaccines [5]–[9]. Since many potential vaccinees including children in the WHO Expanded Program for Immunization reside in areas with high rates of malaria, it is important to understand the effect of malaria infections on the immunogenicity and effectiveness of vaccines designed to prevent tuberculosis.

To combat the lethal tuberculosis epidemic, numerous novel vaccine preparations and immunization strategies are being created to replace or augment the current TB vaccine, *M. bovis* BCG. While BCG does induce protection against disseminated tuberculous disease in children, it has been relatively ineffective in preventing the most prevalent form of the disease, adult pulmonary TB [10], [11]. Furthermore, vaccination with live BCG poses a considerable risk of serious infection when it is given to infants perinatally infected with HIV [12]–[14]. Among the new TB vaccine types being tested to replace or augment the use of BCG are live, attenuated *M. tuberculosis* vaccines, TB fusion proteins formulated in immunostimulating adjuvants, and viral vectored vaccines. At least 10 of these new vaccine preparations are currently being evaluated in clinical trials [15]–[17]. While the efficacy of each of these new vaccine formulations have been assessed in pre-clinical *M. tuberculosis* vaccination/challenge models, the new TB vaccines have been only minimally evaluated in co-infection models. Despite the considerable public health importance of concomitant infections, the complex issues associated with developing immunity after immunization in the presence of co-infecting organisms generally have not been adequately addressed. To develop more efficacious therapeutic and vaccination strategies, it is imperative to dissect whether effective protective immune responses can be generated against deadly pathogens in individuals co-infected with multiple organisms. In particular, it is uncertain whether a malaria infection will alter the effectiveness of new candidate vaccines to protect against a tuberculous challenge. Given the documented immunsuppressive capacity of the malaria parasite, the potential inhibitory impact of malaria infections against the protective immunity induced by new TB vaccines is a significant concern.

Although concurrent helminth or HIV infections have been shown to suppress BCG-induced anti-tuberculosis protective responses, the effect of malaria co-infections on the protective efficacy of vaccines designed to protect against tuberculosis has not been thoroughly investigated [18]–[20]. In this study, we examined the impact of malaria co-infections on the capacity of BCG and new TB vaccines to protect against an aerogenic virulent *M. tuberculosis* challenge of mice. The *P. yoelii* 17XNL parasite was used as a source of malaria infection in mice. The effect of the malaria infection on the immunity induced by TB vaccines was assessed *in vitro* using flow cytometry and *in vivo* with a standard mouse model of pulmonary tuberculosis. Although the flow cytometric data suggest that specific vaccine-induced immune responses can be suppressed by acute malaria infections, no overall reduction in pulmonary protection against TB was detected in vaccinated co-infected mice.

## Materials and Methods

### Animals

C57BL/6 female mice that were 6–8 weeks of age were obtained from the Jackson Laboratories (Bar Harbour, Maine). All mice used in this study were maintained under appropriate conditions at the Center for Biologics Evaluation and Research, Bethesda, MD. This study was done in accordance with the guidelines for the care and use of laboratory animals specified by the National Institutes of Health. This protocol was approved by the Institutional Animal Care and Use Committee of the Center for Biologics Evaluation and Research under Animal Study Protocol 1993-09.

### Vaccines

The BCG Pasteur vaccine preparation was derived from the mycobacterial culture collection of the Trudeau Institute. The E6-85B protein is an ESAT6-antigen 85B *M. tuberculosis* fusion protein which was purified by nickel affinity chromatography after cloning and expressing the ESAT6-antigen 85B fusion gene in the pET23b vector system (Novagen, SanDiego CA). The protein-adjuvant formulation was prepared by mixing the fusion protein (50 µg/ml) with dimethyldioctadecylammonium bromide (DDA; 150 µg/ml; Kodak) and monophosphoryl lipid (MPL; 250 µg/ml; Avanti Polar Lipids, Alabaster, AL). The MVA-5TB vaccine was generated by cloning five *M. tuberculosis* genes (antigen 85A, antigen 85B, ESAT6, Mtb39 and HSP65) as well as the interleukin-15 (IL-15) gene into a modified vaccinia virus Ankara (MVA) vector [21].The double deletion mutant strain (*ΔsecA2ΔlysA*) of the H37Rv strain of *M. tuberculosis* was constructed using specialized transduction to disrupt the chromosomal copy of the *lysA* gene of an unmarked *ΔsecA2* clone, as described previously [22].

### Immunizations

Five female C57BL/6 mice per group were used in the immunization studies. For live BCG vaccine, 10^6^ CFU was given once subcutaneously. Five micrograms of the E6-85 protein in the DDA (15 µg)–MPL (25 µg) adjuvant was administered three times, 2 weeks apart. For the attenuated strain/protein mixture vaccine, 10^6^ CFU of the *ΔsecA2ΔlysA* live attenuated M. tuberculosis strain was mixed with the E6-85/DDA adjuvant formulation and administered three times, 2 weeks apart. For the prime boost experiments, one month after the three priming vaccinations with the E6-85 vaccine preparation, two doses of 5×10^7^ PFU of the MVA/IL15/5TB construct were given subcutaneously 1 month apart.

#### *Plasmodium yoelii* NL infections

Frozen stocks of *P. yoelii* 17XNL-infected erythrocytes were thawed and used to intra-peritoneally (ip) infect three donor C57BL/6 mice. Percent parasitemias were then monitored every other day using blood smears. When ∼10 to 20% parasitemias were detected, blood was collected by cardiac puncture, diluted in PBS and used to infect experimental animals with 1×10^6^ *P. yoelii* 17XNL parasites in 200 ul of PBS by the ip. route. In these studies, five to fifteen C57BL/6 mice per group were used.

### Evaluation of vaccine-induced protection using a mouse model of pulmonary tuberculosis

For the vaccination/challenge experiments, five mice were evaluated for each group. At 2, 6, or 10 weeks following the *P. yoelii* infections, vaccinated and control mice were aerogenically challenged with the *M. tuberculosis* Erdman suspended in PBS at a concentration known to deliver 100–200 CFU in the lungs over a 30-min exposure time in a Middlebrook chamber (GlasCol, Terre Haute, IN). To assess the level of pulmonary exposure during the aerosol challenge, the number of CFU in the lung were measured at 4 h after the *M. tuberculosis* infection. To determine the extent of pulmonary bacterial growth, the mice were sacrificed at 4 weeks post-challenge. The lungs were then removed aseptically and homogenized separately in PBS using a Seward Stomacher 80 blender (Tekmar, Cincinnati, OH). The lung homogenates were diluted serially in 0.4% PBS–Tween 80, and 50-µl aliquots were placed on Middlebrook 7H11 agar (Difco) plates supplemented with 10% OADC enrichment (BectonDickinson, Sparks, MD) medium, 2 µg/ml 2-thiophenecarboxylic acid hydrazide (TCH) (Sigma), 10 µg/ml ampicillin, and 50 µg/ml cycloheximide (Sigma). The addition of TCH to the agar plates inhibits the growth of BCG but not *M. tuberculosis*. All plates were incubated at 37°C for 14 to 17 days in sealed plastic bags, and the colonies were counted to determine the organ bacterial burdens.

### Assessment of lung inflammation

To evaluate the level of inflammation in the lungs of mice infected with *M. tuberculosis*, lung sections stained with hematoxylin and eosin (H & E) were photographed using a Nikon Optishot 2 microscope fitted with a camera which was connected to a computer. Spot Advanced software was used to save the computer images. The Image Pro Plus program (Media Cybernetics, Silver Spring, MD) was utilized to objectively assess the level of inflammation present in each image. In these images, the inflamed areas stained a more intense purple than the non-inflamed areas. For the analyses, colors were assigned as follows: red to represent the inflamed areas, green to represent non-inflamed areas, and yellow to represent the background. After the color assignments were established, the computer software identified inflamed and non-inflamed sections on each slide. The percentage of the lung sections staining red, green, or yellow was then determined by the computer software. To quantitate the percent area inflamed, we determined the mean percent red area from five lung sections of each of the different groups.

### Flow cytometry

Five BCG vaccinated and control mice (3 mice per group) were used to determine the frequency of CD4 MFT cells at each time point post-vaccination. Lung cells were isolated by homogenizing the lung tissue in a stomacher bag using the end of a 20 cc syringe in PBS containing 2% FBS (PBS-FBS). The tissue was then incubated in PBS-FBS containing 4 mg/ml collagenase (final concentration) at 37°C for one hour. Afterward, the lung tissue was removed from the cells by placing the suspension in a Filtra-Bag (Labplas, Quebec, Canada). The resulting single cell suspension was centrifuged to pellet the cells and treated with ACK lysing buffer as described above. After washing, the cells were passed through a 70 µm cell strainer, pelleted and counted.

After washing the lung cells with an equal volume of media, the cells were resuspended in cDMEM-FBS, counted and added to wells of a 24-well plate at a density of 2.5×10^6^ cells per well in 1.0 ml cDMEM-FBS. For measurement of antigen-specific responses, BCG Pasteur (or BCG+PPD) was added to the wells at a multiplicity of infection (MOI) of 0.5 bacilli per spleen cell. Wells which contained only lung cells served as unstimulated controls. Infections were allowed to proceed overnight followed by the addition of Golgiplug (BD Biosciences, San Jose CA) (1 µl per well). After 4–5 hours of incubation, the unbound cells were removed from the wells and transferred to 12×75 mm tubes, washed with PBS and resuspended in ∼50 µl PBS. Live-Dead stain (Invitrogen, Carlsbad, CA) (10 µl of a 1∶100 dilution) was added to each tube and incubated for 30 min. at room temperature to allow for gating on viable cells. After washing the cells with PBS-FBS, antibody against CD16/CD32 (FcγIII/II receptor, clone 2.4G2) (Fc block) was added in a volume of ∼50 µl and incubated at 4°C for 15 min. The cells were then stained for 30 min. at 4°C by adding antibodies against the CD4 (rat anti-mouse CD4 Alexa Fluor 700 [AF-700] Ab, clone RM4–5), and CD8 (rat anti-mouse CD8 peridinin chlorophyll protein complex [PerCP] Ab, clone 53-6.7) proteins at 0.1 and 0.2 µg per tube respectively. Following the incubation, the cells were washed twice with PBS and then fixed for 30 min. at 4°C with 2% paraformaldehyde in PBS. After fixing, the cells were pelleted, washed twice with PBS-FBS and stored at 4°C. Fixed cells were washed twice with perm-wash buffer (1% FBS, 0.01 M HEPES, 0.1% saponin in PBS) followed by intracellular staining using the following antibodies at 0.2 µg per tube: rat anti-mouse IFN-γ allophycocyanin [APC] Ab, clone XMG1.2; rat anti-mouse TNF-α fluorescein isothiocyanate [FITC] Ab, clone MP6-XT22; rat anti-mouse IL-2 phycoerythrin [PE] Ab, clone JES6-5H4. The cells were incubated at 4°C for 30 min., washed twice with perm-wash buffer and then twice with PBS-FBS. All antibodies were obtained from BD Biosciences.

The cells were analyzed using a LSRII flow cytometer (Becton Dickinson) and FlowJo software (Tree Star Inc., Ashland, Oregon). We acquired 250,000 events per sample and then, using FlowJo, gated on live, single cell lymphocytes. To determine the frequency of different populations of MFT cells, we gated on CD4 or CD8 T cells staining positive for TNF-α and IFN-γ, TNF-α and IL-2, IFN-γ and IL-2 or all three cytokines.

### Median fluorescence intensity (MFI) assessments

The MFI for IFN-γ or TNF-α from monofunctional and multifunctional CD4 and CD8 T cells was evaluated using the FlowJo software. For this study, the MFI is the average fluorescence intensity value for individual T cells secreting only IFN-γ or TNF-α, secreting both IFN-γ and TNF-α or cells secreting IFN-γ, TNF-α and IL-2. The data are presented as the mean ± the standard error of the individual MFI assessments for 5 groups of mice.

### Statistical analyses

The protection, lung inflammation, and flow cytometry data were evaluated using t test analysis of the GraphPad Prism, version 5, program.

## Results

### The impact of malaria co-infections on the effectiveness of BCG vaccine in a mouse model of pulmonary tuberculosis

To assess the impact of a malaria co-infection on the effectiveness of vaccines designed to protect against *M. tuberculosis*, a murine co-infection immunization model was developed. Initially, mice were vaccinated subcutaneously with 10^6^ CFU of BCG Pasteur. Two months after the BCG immunization, the mice were given 10^6^ *P. yoelii* blood stage parasites by the intraperitoneal route. At an appropriate time period following the malaria infection (2–10 weeks), the mice were aerogenically challenged with 100–200 CFU of virulent *M. tuberculosis* Erdman. Four weeks later, the TB infected mice were sacrificed and pulmonary mycobacterial burdens and lung pathologies were determined. For these studies, since extensive splenomegaly was seen after malaria infections and the lung is the primary site of *M. tuberculosis* infections, we concentrated on evaluating the pulmonary impact of the co-infection on the effectiveness of TB vaccines.

**Figure 1:**
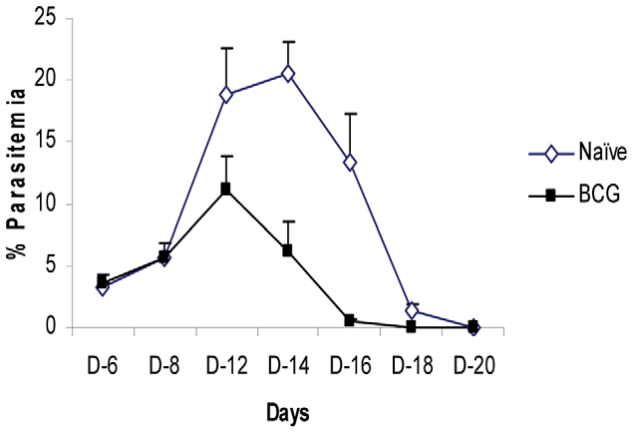
The levels of parasitemias following a *P. yoelii* infection of naïve and BCG-vaccinated C57BL/6 mice. The parasitemia levels were detected in blood smears from 5–10 BCG vaccinated (closed squares) and naïve (open triangles) mice at a specific day after the *P. yoelii* challenge. The parasitemia curves contain data from a single experiment which is representative of results of the *P. yoelii* infections done in this study.

Since our aerosol TB challenge model had been previously established, our initial efforts for these studies focused on characterizing the kinetics of the *P. yoelii* infection. In the representative data shown in Figure 1, malaria parasitemia in naïve mice peaked at 20.5% on day 14 and the infection was cleared by day 20. Consistent with previous studies, moderate protection against malaria parasitemia was seen in BCG vaccinated mice [23], [24]. In this experiment, the level of parasitemia was reduced by 41% relative to naives at day 12 and 70% at day 14 in BCG-vaccinated mice but parasite clearance was again seen by day 20.

**Figure 2:**
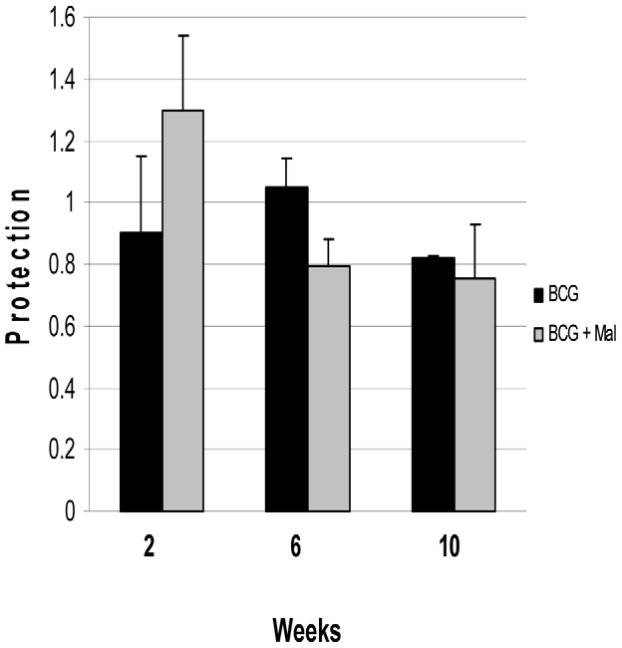
Infection with *Plasmodium yoelii* NLmalaria does not reduce the anti-tuberculosis pulmonary protective immunity induced by immunization with BCG vaccine. C57Bl/6 mice were vaccinated subcutaneously with BCG and two months later infected with *P. yoelii* by the intraperitoneal route. The mice were challenged by the aerosol route with *M. tuberculosis* Erdman at 2, 6, or 10 weeks after the infection with *P. yoelii*. Mycobacterial CFU in the lung were assessed at 4 weeks post-challenge. The data are represented as the mean (± the standard error of the mean) protection seen in BCG-vaccinated non-infected (black bars) and BCG-vaccinated *P. yoelii* infected mice (grey bars). Protection is defined as the lung CFU (log_10_) difference between naïve and BCG-vaccinated mice.

The initial vaccination/challenge studies evaluated the temporal effect of *P. yoelii* infections on the effectiveness of BCG vaccine to protect against an aerogenic *M. tuberculosis* challenge. Mice were infected with *P. yoelii* two months after BCG immunization and then were challenged with *M. tuberculosis* at either two, six or ten weeks following the malaria infection. When mice were challenged with *M. tuberculosis* at two weeks after the malaria infection (at peak parasitemia levels), no significant impact was detected on the capacity of naïve or BCG vaccinated mice to control the acute tuberculosis lung infections at 4 weeks post-challenge (Figure 2). In both malaria infected and non-infected BCG vaccinated mice, significant anti-tuberculosis protection (>0.95 log_10_ compared to naïve controls) were seen at 4 weeks after the *M. tuberculosis* challenge. Similarly, at 6 and 10 weeks following the malaria infection, the anti-tuberculosis protective responses induced by the BCG vaccinated mice were not statistically different than the protection evoked in *P. yoelii* infected BCG vaccinated animals. Additionally, the pulmonary mycobacterial burdens were also not different in the naïve and *P. yoelii* infected naïve groups at 2, 6 and 10 week post malaria infection time point (data not shown). For example, the lung CFU levels that were detected when non-vaccinated mice were challenged with *M. tuberculosis* at the peak of malaria parasitemia (6.36±0.12) were statistically equivalent to lung burdens seen in naïve mice infected with *M. tuberculosis* (6.26±0.18). Overall, the presence of malaria parasites did not exacerbate the tuberculous pulmonary infection when the *M. tuberculosis* challenge occurred either near the peak of parasitemia or after parasite clearance. Interestingly, nearly identical results were obtained in co-infection studies of mice that had been vaccinated with BCG eight months before the *P. yoelii* blood stage infection and then were challenged with *M. tuberculosis* at the peak of parasitemia. At 8 months after BCG immunization, statistically equivalent lung CFU levels were detected in the BCG (5.59±0.11 log_10_ CFU) and BCG/P. *yoelii* (5.58±0.22) groups as well as the naïve (6.40±0.30) and naïve/*P. yoelii* (6.11±0.15) animals.

To support these findings, lung pathology was analyzed using H&E sections from mice that were challenged with *M. tuberculosis* two weeks after a *P. yoelii* infection. Overall, the *P. yoelii* infections did not impact lung pathology observed after an aerogenic *M. tuberculosis* infection. At 4 weeks post challenge, substantially less inflammation was observed in lung sections of BCG vaccinated and the BCG/*P. yoelii* infected animals relative to naïve controls (Figure S1). The granulomatous type structures were more condensed, mature, and lymphocyte-rich in the lungs of both BCG vaccinated groups compared to the larger, more immature granulomas seen in the naïve and naïve/*P. yoelii* mice at this time point. To quantitate the pathology results, the lung sections were assessed by computerized scanning using the Image pro analysis system as described earlier [25]. With this imaging system, the proportion of lung sections that are inflamed can be quantitatively defined. This pathology analysis showed no statistical differences in the inflammatory response values seen in lung sections taken from the BCG (20.2±6.4) and BCG/*P. yoelii* infected mice (20.9±9.0). Similarly, the lung pathology values were not different in naïve (38.6±10.6) and naïve/P. *yoelii* infected (34.9±3.9) animals.

### The impact of malaria-*M. tuberculosis* co-infections on the protective immunity induced by novel TB vaccines

**Figure 3:**
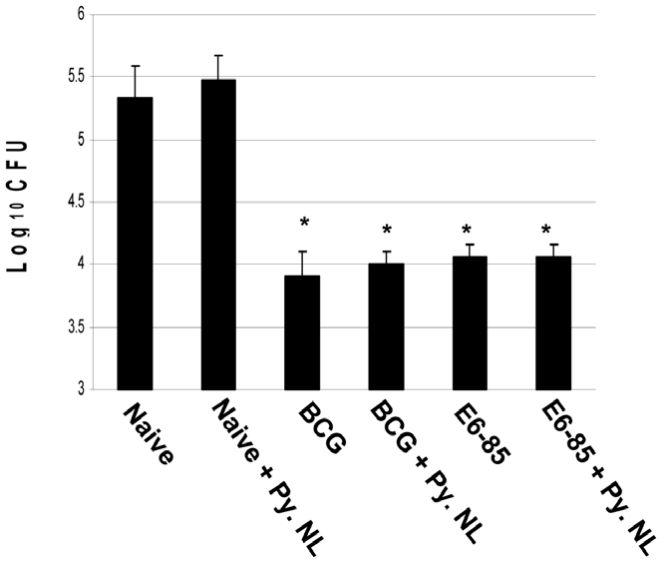
Infection with *P. yoelii* malaria does not decrease the acute anti-tuberculosis pulmonary protection induced by immunization with the *M. tuberculosis* ESAT6-Antigen 85 fusion protein formulated in DDA/MPL adjuvant. C57Bl/6 mice were vaccinated once with BCG or three times two weeks apart the E6-85/adjuvant vaccine and were infected with *P. yoelii* two months after the initial vaccination. The mice were aerogenically challenged with virulent *M. tuberculosis* at 2 weeks after the *P. yoelii* infection and pulmonary mycobacterial CFU were detemined at 4 weeks post-challenge. The asterisks show significant CFU differences (p<0.05) relative to naïve controls.

**Figure 4:**
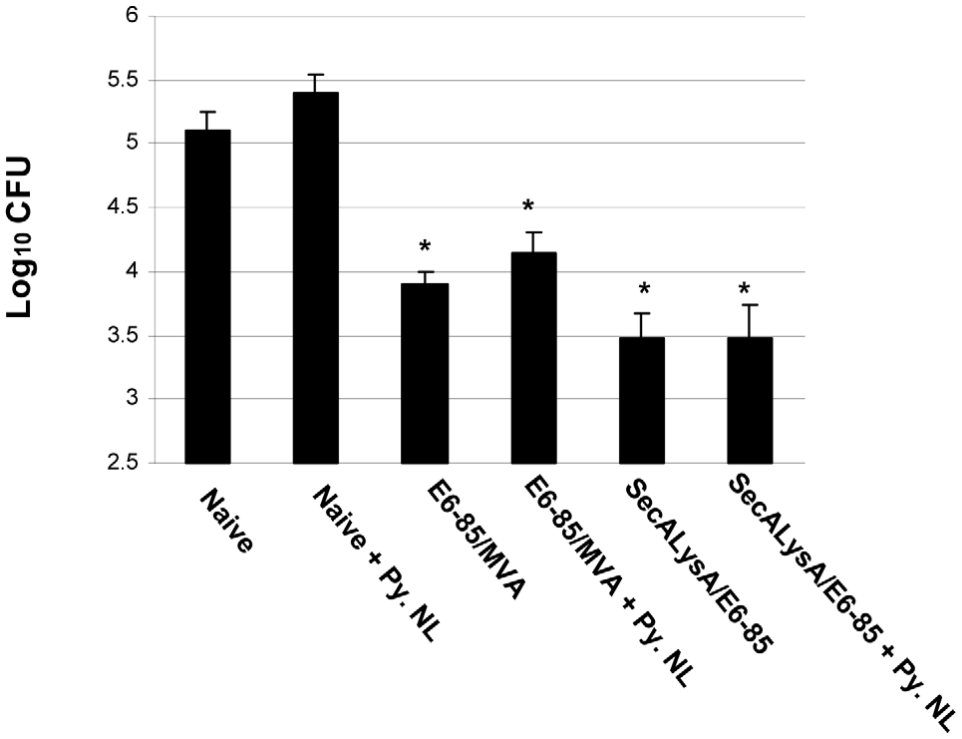
Infection with *P. yoelii* malaria does not decrease the acute anti-tuberculosis pulmonary protection induced by vaccination with the *ΔsecAΔlysA* attenuated *M. tuberculosis* strain mixed with the E6-85/adjuvant formulation or immunization with the E6-85/adjuvant followed by boosting with TBMVA/IL-15 vaccine. For the *ΔsecAΔlysA* mixture vaccine, C57Bl/6 mice were vaccinated three times two weeks apart and then were infected with *P. yoelii* 4 months after the final vaccination. For the prime/boost protocol, mice were vaccinated with the E6-85/adjuvant formulation three times two weeks apart, and one month later boosted with the TBMVA/IL-15 vaccine twice one month apart. The mice that had been primed and boosted were infected with *P. yoelii* two months after the final booster vaccination. All vaccinated mice were aerogenically challenged with virulent *M. tuberculosis* at 2 weeks after a *P. yoelii* infection and pulmonary mycobacterial CFU were determined at 4 weeks post-challenge. The asterisks show significant CFU differences (p<0.05) relative to naïve controls.

To assess the impact of malaria infection on the effectiveness of novel TB vaccine candidates, C57BL/6 mice were vaccinated with three unique immunizing preparations using different vaccination strategies. In an initial experiment with a novel vaccine, mice were immunized with the E6-85 TB fusion protein (ESAT6-Antigen 85B) suspended in DDA/MPL adjuvant [26], [27]. As controls, other groups of mice were immunized with BCG. At two weeks after a *P. yoelii* infection, mice were aerogenically challenged with a low dose of *M. tuberculosis*. Four weeks later, pulmonary bacterial burdens were evaluated. Again no significant differences in lung CFU were seen between the malaria-infected and the non-infected control groups. As seen in Figure 3, the *P. yoelii* infection clearly did not increase pulmonary *M. tuberculosis* CFU levels in non-vaccinated mice. Moreover, the levels of protection detected in the vaccinated animals were consistent with previous results and were unaffected by the *P. yoelii* infection (1.4 log_10_ for the BCG and BCG/*P. yoelii* groups; 1.2 log_10_ for the E6-85 and E6-85/*P. yoelii* mice) [26]. In a second study of novel TB vaccination strategies, mice were immunized with either a attenuated *M. tuberculosis ΔsecAΔlysA* vaccine strain mixed with the E6-85/DDA formulation or a prime-boost procedure that involved priming with the E6-85 protein adjuvant mixture followed by boosting with a MVA-based vaccine which over-expressed 5 *M. tuberculosis* antigens and IL-15 (MVA/IL-15/5Mtb) [28], [29]. As shown in Figure 4, when the *M. tuberculosis* challenge occurred during elevated levels of parasitemia (2 weeks), the extent of anti-tuberculosis protection induced by the attenuated *M. tuberculosis* vaccine/protein mixture and the prime-boost immunization procedure were not impacted by the malaria infection. About 1.3 log_10_ protection was seen in both malaria infected and non-infected prime-boost groups and 1.6–1.7 log_10_ protective responses were measured for both the *ΔsecAΔlysA M. tuberculosis* attenuated vaccine mixture and *ΔsecAΔlysA* vaccine/*P. yoelii* infected animals Furthermore, the malaria infection again did not exacerbate the tuberculous disease because no significant differences in pulmonary mycobacterial burdens were again detected between the malaria infected non-vaccinated and naïve control groups at 4 weeks post challenge.

### Flow cytometric analysis of vaccine-induced immune responses in BCG vaccinated and malaria infected BCG vaccinated mice

**Figure 5:**
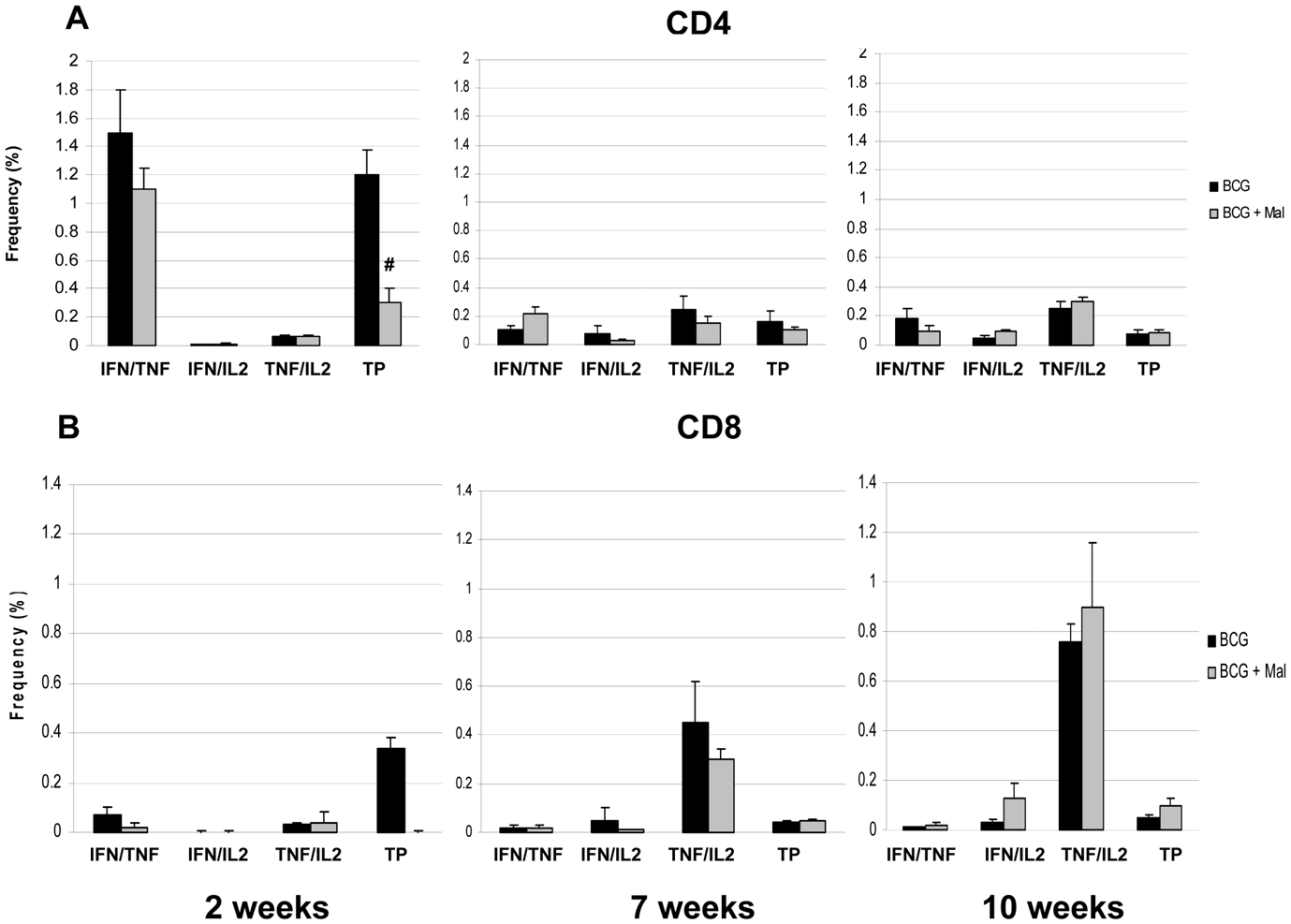
The frequency of CD4 (A) and CD8 (B) MFT cells recovered form the lungs of BCG vaccinated (black bars) and BCG vaccinated, malaria infected (grey bars) mice at 2, 7, and 10 weeks following the *P. yoelii* challenge. Lung cells were removed and pooled from 3 mice per group, stimulated overnight with BCG, and analyzed by multi-parameter flow cytometry to determine the frequency of cells producing either IFN-γ and TNF-α (IFN/TNF), IFN-γ and IL-2 (IFN/IL2), TNF-α and IL-2 (TNF/IL2), and IFN-γ, TNF-α, and IL-2 (Triple Positive, TP). The data are presented as the mean frequency ± SEM for 5 groups of mice. #, Significant differences between the cellular frequencies of the BCG vaccinated and the BCG vaccinated, malaria infected groups.

Recent studies have shown that malaria infections can have immunomodulatory effects on host immune responses and in particular, malaria can inhibit antigen specific T cell responses [5]–[9]. To assess whether pulmonary mycobacterial-specific T cell responses were influenced by the malaria infection in our model, multi-parameter flow cytometric analysis was done on lung cells recovered from experimental animals. For these studies, mice were infected with *P. yoelii* two months after BCG vaccination and then 2, 7, or 10 weeks later the mice were sacrificed and the lung cells were isolated and stimulated with BCG (a surrogate for the *M. tuberculosis* challenge). Following intracellular cytokine staining, the cells were analyzed by flow cytometry. Since the induction of multifunctional T cells (MFT) by immunization has been shown to correlate with protection against Leishmania and *M. tuberculosis* in animal models, the cells were evaluated for the concurrent expression of IFN-γ TNF-α and/or IL-2 [30]–[32]. As seen in Figure 5A and Figure S2, the levels of CD4 cells producing IFN-γ, IFN-γ/TNF-α, or IFN-γ/TNF-α/IL2 all exceeded 1% in BCG vaccinated mice at week 2 of the *P. yoelii* infection (10 weeks post-BCG immunization) but declined at weeks 7 and 10 post-infection (15 and 18 weeks post-BCG vaccination). In contrast, the frequency of IL-2 and TNF-α/IL2 producing cells in BCG vaccinated animals significantly increased during the 10 week observation period. In these studies, the frequencies of cells from naïve controls expressing multiple cytokines were generally less than 0.01% (data not shown). Although lower overall cell frequencies were seen, a similar pattern was observed for CD8 T cells taken from BCG vaccinated animals that were not infected with malaria (Figure 5B). The relative proportions of cells producing IFN-,γ IFN-γ/TNF-α and IFN-γ/TNF-α/IL2 were elevated at 10 weeks after the BCG immunization while the frequencies of these cells declined in the lung 5–7 weeks later. Consistent with the CD4 data, the frequency of IL-2 and TNF-γ/IL2 producing CD8 T cells increased at the later time points of the experiment, but the magnitude was higher than that seen for CD4 cells.

Surprisingly, the malaria infection did not generally impact the frequencies of vaccine-induced CD4 and CD8 cytokine producing cells at the later stages of this study. At 7 and 10 weeks after the malaria challenge (15 and 17 weeks post-BCG vaccination), malaria-related alterations in the cellular frequencies were not observed in BCG vaccinated animals. However, a negative impact was seen on the frequencies of cells synthesizing IFN-γ/TNF-α/IL2 in BCG vaccinated mice when malaria parasitemias were substantially elevated (2 weeks post *P. yoelii* infection). For the CD4 T cells, the frequencies of triple positive cells were significantly decreased (BCG = 1.22%, BCG/*P. yoelii* = 0.29%). Interestingly, dramatic declines in the triple positive CD8 T cells were also seen at the peak of *P. yoelii* infection (BCG = 0.355%, BCG/*P.yoelii* = 0.002%).

**Figure 6:**
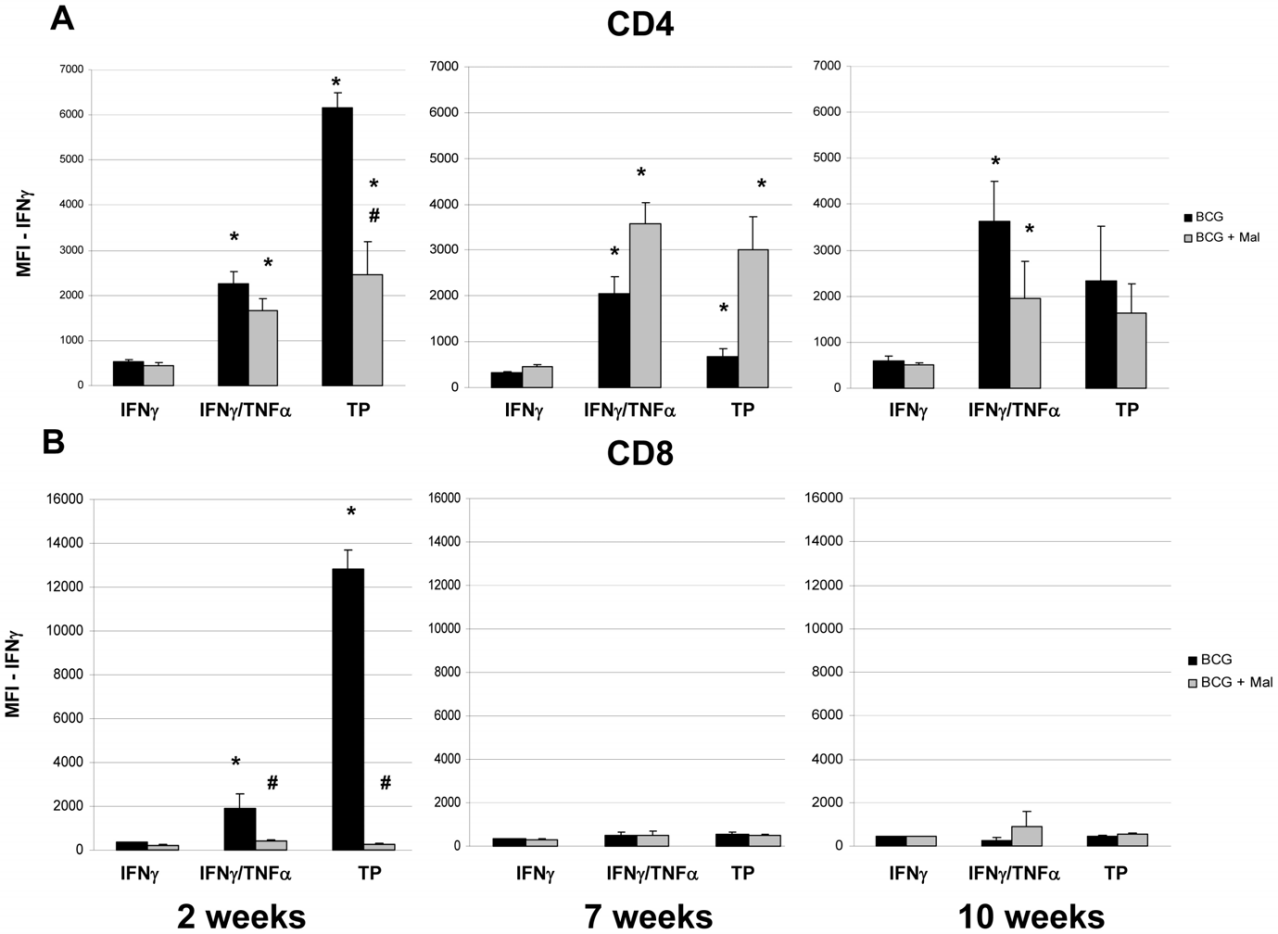
The Median Fluorescent Intensities (MFI) for IFN-γ of CD4 (A) and CD8 (B) T cells recovered from the lungs of BCG-vaccinated (black bars) and BCG vaccinated, malaria infected (grey bars) mice at 2, 7, and 10 weeks following a *P. yoelii* challenge. The lung cells were pooled from 3 mice per group, stimulated overnight with BCG, and analyzed by flow cytometry. The MFIs are presented as the mean MFI ± SEM for 5 groups. IFN = monofunctional IFN-γ producing T cells, IFN/TNF = multifunctional IFN-γ and TNF-α expressing T cells, Triple Positive (TP) = IFN-γ, TNF-α, and IL-2 producing T cells. * MFI values for IFN-γ that are significantly higher than the monofunctional cells. # IFN-γ MFI responses which are significantly lower in the BCG vaccinated, malaria-infected mice compared to BCG vaccinated controls.

An important characteristic of MFT cells is their capacity to express substantially higher levels of cytokines than monofunctional cells. To further evaluate the effect of malaria infections on the immune responses induced by BCG vaccinated mice, the levels of cytokine production in pulmonary CD4 and CD8 T cells was assessed. For this study, the extent of cytokine expression was determined by evaluating the median fluorescence intensities (MFI) of the experimental lung cells. In contrast to the cellular frequencies of pulmonary cells from BCG vaccinated mice, IFN-γ MFI values for CD4 MFT cells remained elevated throughout the study. As expected, the levels of IFN-γ expressed in IFN-γ/TNF-α and triple positive CD4 MFT cells from BCG vaccinated mice were increased 4–11 fold (relative to monofunctional IFN-γ producing CD4 T cells) during the entire study. Although the IFN-γ MFI values for CD4 MFT cells from BCG immunized mice were not effected at 7 and 10 weeks after the malaria challenge, the extent of IFN-γ expression in triple-positive CD 4 T cells was reduced 60% in the malaria infected, BCG vaccinated animals compared to the BCG vaccinated controls at 2 weeks post *P. yoelii* challenge (Figure 6). While the impact of BCG vaccination was minimal at the later time points, substantially elevated IFN-γ MFI values were detected in CD8 T cells at the 2 week time point. Relative to monofunctional cells, increased IFN-γ MFI values (5–35 fold) were detected by flow cytometric analysis of IFN-γ/TNF-α and IFN-γ/TNF-α/IL2 producing cells at the early time point. Interestingly, significantly suppressed MFI values were seen in CD8 MFT cells recovered from malaria infected, BCG vaccinated mice at the peak of the *P. yoelii* infection. In these mice, the IFN-γ MFI values were decreased by 79% (IFN-γ/TNF-α CD8 cells) and 98% (triple positive CD8 cells) by the malaria infection.

**Figure 7:**
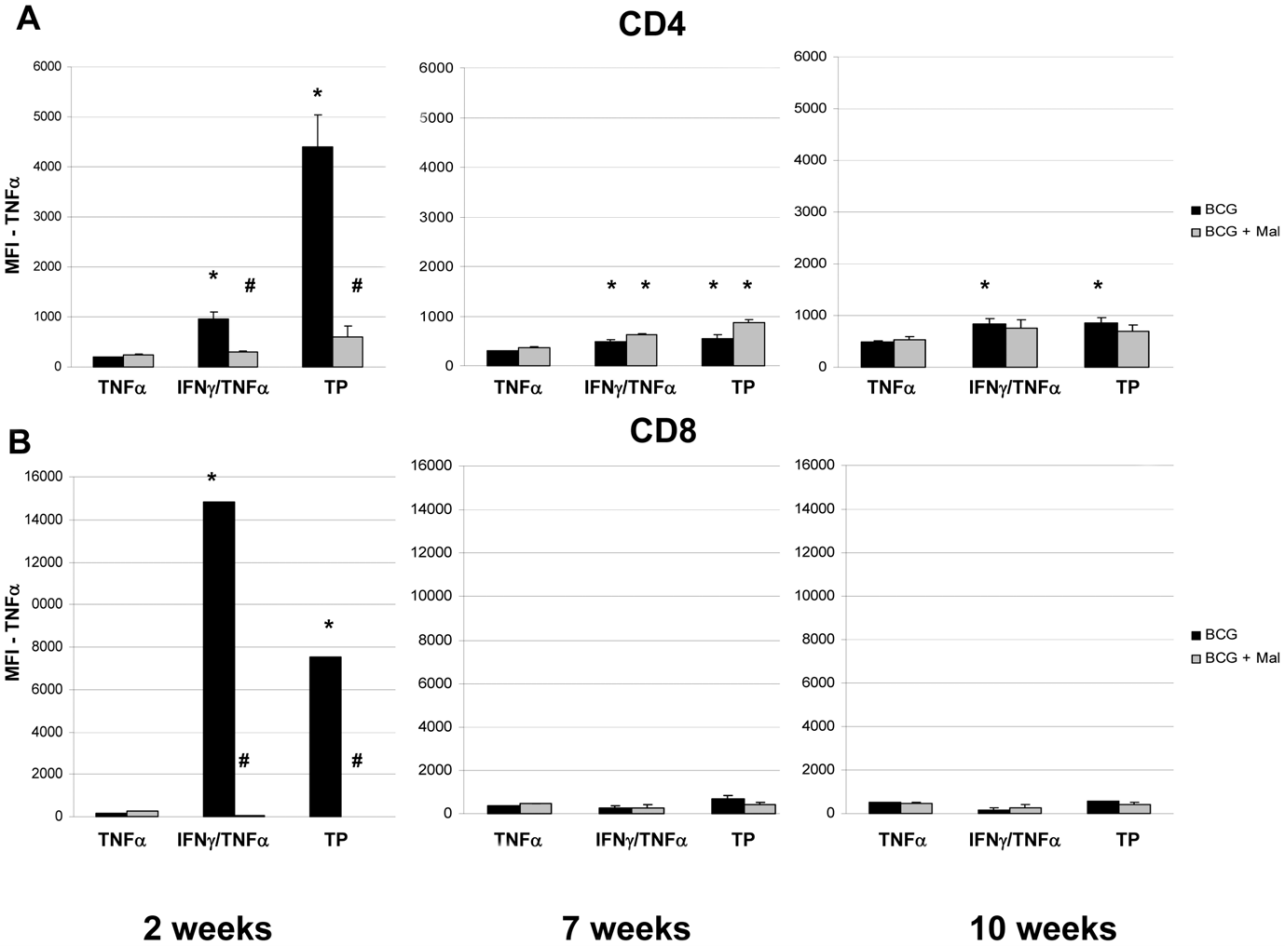
The Median Fluorescent Intensities (MFI) for TNF-α of CD4 (A) and CD8 (B) T cells recovered from the lungs of BCG-vaccinated (black bars) and BCG vaccinated, malaria infected (grey bars) mice at 2, 7, and 10 weeks following a *P. yoelii* challenge. The lung cells were pooled from 3 mice per group, stimulated overnight with BCG, and analyzed by flow cytometry. The MFIs are presented as the mean MFI ± SEM for 4 groups. TNF = monofunctional TNF-α producing T cells, IFN/TNF = multifunctional IFN-γ and TNF-α expressing T cells, TP (Triple Positive) = IFN-γ, TNF-α, and IL-2 producing T cells. * MFI values for TNF-α that are significantly higher than the monofunctional cells. # TNF-α MFI responses which are significantly lower in the BCG vaccinated, malaria-infected mice compared to BCG vaccinated controls.

Similarly, elevated TNF-α MFI values for CD4 T cells of BCG vaccinated mice were detected in IFN-γ/TNF-α double positive (increased 5 fold compared to monofunctional cells) and triple positive MFT cells (22 fold increase) at the two week time point (Figure 7). In contrast, for the malaria infected, BCG vaccinated mice, the TNF-α MFI values of CD4 T cells were strikingly reduced in IFN-γ/TNF-α (70% reduction) and triple positive cells (89%) relative to controls not infected with *P. yoelii*. While significant 2–3-fold increases in TNF-α MFI values compared to controls were seen in CD4 MFT cells at the later time points, the malaria infections did not impact the lower overall MFI values. For CD8 cells, substantially elevated TNF-α MFI values relative to monofunctional cells were observed for the IFN-γ/TNF-α (88x) and the triple positive cells at 2 weeks (44x) after the malaria infection. However, for the malaria infected, BCG vaccinated mice, dramatic declines in CD8 TNF-α MFI values of >99% were detected in IFN-γ/TNF-α and triple positive CD8 MFT cells at two weeks after the *P. yoelii* infection. At 7 and 10 weeks post-infection, consistently low TNF-α MFI values were seen in all CD8 T cell subsets.

## Discussion

An understated concern about the deployment of new TB vaccines is the unknown impact that infections with other pathogens prevalent in the area may have on TB vaccine efficacy. In many areas endemic for tuberculosis, co-infections with unrelated pathogens are common and these co-infecting agents may modulate vaccine-induced immune responses. Earlier studies have shown that concurrent infections can decrease the anti-tuberculosis protective responses induced by immunization with BCG. In animal models, helminth infections have been shown to reduce the efficacy of BCG vaccine to protect against virulent *M. tuberculosis* [18]. Exposure to non-tuberculous mycobacteria can also inhibit the induction of protective immunity to tuberculosis by BCG immunization [33], [34]. In humans, HIV infection can severely impair the protective immune responses elicited by vaccination with BCG [20]. With the considerable geographic overlap in areas endemic for malaria and tuberculosis and the recent reports of co-infection of these organisms, it is important to assess the impact of malaria infections on the effectiveness of vaccines designed to prevent tuberculosis in pre-clinicalanimal models. Many different animal species are susceptible to tuberculous infections and artificially infected mice, guinea pigs, rabbits, and non-human primates (NHP) have been used as models of TB [35]. While mice (like humans) are relatively resistant to TB, can be infected by the aerosol route, and have been successfully used to elucidate host-pathogen interactions, TB infections of guinea pigs and rabbits yield more relevant lung pathology. Although the NHP model has been valaubale for studying TB latency as well as host immune responses, the cost, BSL-3 space requirements, and the potential for horizontal transmission of disease has limited its usefulness. Given that we had previously established standardized and affordable murine models of TB and malaria we decided to develop a mouse TB-malaria co-infection model. In this study, we showed using this mouse model that *P. yoelii* malaria co-infections did not have a significant impact on the capacity of four different *M. tuberculosis* vaccine formulations to control pulmonary growth of an acute virulent *M. tuberculosis* infection. In repeated experiments, we demonstrated that the pulmonary protective responses induced by vaccination with either BCG, the E6-Ag85 TB fusion protein formulated in adjuvant, a *ΔsecAΔlysA M..tuberculosis* attenuated strain/protein mixture or a prime-boost strategy involving the E6-85 antigen preparation and the MVA/IL15/5Mtb vaccine were not statistically different in immunized mice that had been infected with *P. yoelii* relative to uninfected vaccinated controls. These findings are consistent with results from a malaria chemoprophylaxis trial of Nigerian children where the immunogenicity of BCG vaccine was not affected by the presence of malaria parasitemia [6]. Collectively, these data suggest that a primary infection with malaria parasites will likely not significantly impact the capacity of new TB vaccines to control acute *M. tuberculosis* infections in humans.

Although malaria infections in a murine model did not reduce the overall protection in the lung induced by vaccination against TB, BCG vaccine-induced pulmonary immune responses were impacted by elevated malaria parasitemia levels. Published reports in humans and mice have shown that CD4 and CD8 T cell responses against malaria or non-malaria antigens can be inhibited by malaria infections [9], [36]–[39]. In our study, malaria infections were shown to significantly decrease the frequency of CD4 and CD8 triple positive MFT cells expressing IFN-γ, TNF-α, and IL-2 in lung cells of BCG vaccinated mice when high levels of parasitemia were present. Moreover, substantial reductions in cytokine expression (as measured by the median fluorescence intensity) was seen in lung MFT cells from *P. yoelii* infected BCG vaccinated mice relative to uninfected BCG immunized animals. For example, the IFN-γ MFI values decreased by 60% in CD4 triple positive T cells (compared to controls) while a dramatic 98% reduction in MFI values was detected for the CD8 triple positive MFT cells recovered from the lungs of *P. yoelii* infected and BCG vaccinated mice. Additionally, the TNF-α MFI values for CD8 MFT cells was dramatically decreased by 99%. While a substantial suppression of specific T cell responses at 2 weeks after the malaria infection were clearly seen, the mechanisms by which the *P. yoelii* infections reduce the activity of these specific T cell subsets are uncertain. During acute blood stage malaria infections, regulatory T cells producing IL-10 and dendritic cells secreting TGF-β and prostaglandin E2 have been identified [9], [40]. IL-10, TGF-β, and PGE2 have been shown to down-regulate general pro-inflammatory T cell responses, especially CD8 T cell responses. Whether these immune mediators specifically target vaccine-induced MFT cells is currently unclear. Importantly, the activity of MFT cells was not substantially impacted by the *P. yoelii* infection at 7 and 10 weeks after the malaria challenge. At these time points, when the malaria parasitemia in the blood was undetectable, both the cellular frequencies and the MFI values of CD4 and CD8 T cell subsets of the BCG vaccinated and the BCG vaccinated, malaria infected mice were not significantly different. Our data are consistent with the results of an earlier study which showed a substantial recovery of CD8 T cell function at one month after a *P. yoelii* infection [9]. Taken together, these data suggest that the suppression of BCG-induced T cell function by a *P. yoelii* infection is short-lived and the malaria-induced suppressive activity wanes after parasite clearance. It should be noted that a temporal decline in the frequency of IFN-γ, IFN-γ/TNF-α, and triple positive pulmonary T cells was generally observed in BCG vaccinated mice with or without concurrent *P. yoelii* infections. These reduced T cell frequencies seen at 7 and 10 weeks likely resulted because of the declining numbers of BCG organisms in the lung at 4–5 months after the BCG immunizations [41].

The reduction in CD4 and CD8 triple positive MFT responses seen in malaria infected animals at 2 weeks after the *P. yoelii* infection was surprising because of the previously reported correlation between vaccine-induced triple-positive cells and protective immunity. In animal models of *Leishmania* and *M. tuberculosis*, vaccine-induced immune responses from triple positive MFT cells have correlated with in vivo protection against an infectious challenge [30]–[32]. While CD8 T cells are probably not critical for controlling an acute tuberculous infection in mice, CD4 T cells are clearly essential for limiting the proliferation of the pathogen in the lung after an aerogenic *M. tuberculosis* challenge [42], [43]. If there is a linear correlation between the early induction of triple positive CD4 MFT cells by vaccines and anti-tuberculosis protection, then the decreased frequencies and intensities of CD4 MFT triple positive cells observed in the flow cytometric studies of BCG vaccinated malaria infected mice at 2 weeks post-infection should have resulted in decreased protection. The apparent lack of correlation between the early levels of vaccine-induced triple positive responses and anti-tuberculosis protection could have been caused by the absence of malaria-induced suppression seen at later time points in the study when pulmonary bacterial burdens were evaluated. Alternatively, the association between vaccine-induced MFT cell responses and anti-microbe protection may be more complex than has been anticipated. Using a SIV macaque model, Sui et al recently reported that the levels of antigen-specific CD8 MFT cells correlated with protection but the correlation was non-linear and involved a threshold-like effect [44]. In studies of *M. tuberculosis* vaccines, our group recently showed that the anti-tuberculosis protective responses evoked by immunization were also related to the induction of double-positive IFN-γ/TNF-α expressing CD4 T cells [32]. In the current study, the activity of double-positive CD4 MFT cells (which were not suppressed by the malaria infection) could have partially compensated for the reduction of triple-positive CD4 MFT cells seen at two weeks after the malaria challenge. Clearly more studies including well designed longitudinal experiments are needed to delineate the role of vaccine-induced MFT cells in protecting against tuberculous disease. Improved strategies to efficiently purify MFT cells would facilitate studies focusing on the function of these vaccine-induced MFT cells.

An important concern relevant to malaria and tuberculosis co-infections is whether cellular immunosuppression often associated with malaria parasitemia could result in increased cases of clinically detectable tuberculosis. In this study, primary *P. yoelii* infections did not exacerbate acute *M. tuberculosis* lung disease in non-vaccinated mice. In repeated experiments, no statistical differences were seen in pulmonary mycobacterial burdens or lung pathology at one month post-challenge in infected mice relative to naïve controls. In earlier studies, Scott et al and Hawkes et al had reported that malaria exacerbates mycobacterial disease in acute and latent infection models [45], [46]. However, in both studies only modest increases in organ CFU levels and/or survival rates were detected. To further examine the impact of *P. yoelii* parasitemia on tuberculous disease, we are currently evaluating whether malaria infections increase the reactivation rate of mice with low level latent-like TB infections. The results of this study could be helpful for delineating whether malaria infections can contribute to the reactivation of latent tuberculosis.

Overall, our studies in the mouse model of pulmonary tuberculosis suggest that primary malaria co-infections should not significantly impact the efficacy of novel immunization strategies against tuberculosis. However, to confirm these findings, well-designed studies are needed in humans to better understand the complex interactions between these co-infecting organisms. The results of these studies should facilitate the design of more effective immunization and therapeutic procedures against tuberculosis for use in regions with high rates of concomitant infections.

## Supporting Information

Figure S1 **H & E stained lung sections from BCG vaccinated and malaria infected mice after a M. tuberculosis challenge by the aerosol route.** Sections were obtained from naïve, BCG vaccinated, non-immunized-malaria infected and BCG vaccinated-malaria infected mice at 4 weeks after an aerogenic challenge with *M. tuberculosis* and analyzed by computer scanning using an Image pro analysis system. This analaysis showed no statistical diiferences in the inflammatory responses for BCG (20.2±6.4) and BCG/*P. yoelii* infected mice (20.9±9.32). Similarly, significant differences were not seen between the lung pathology values for naïve (38.6±10.6) and naïve/P. yoelii infected (34.9±3.9) animals.
(TIF)

Figure S2 **The frequency of CD4 (A) and CD8 (B) monofunctional cells recovered form the lungs of BCG vaccinated (black bars) and BCG vaccinated, malaria infected (grey bars) mice at 2, 7, and 10 weeks following the** ***P. yoelii*** **challenge.** Lung cells were removed and pooled form 3 mice per group, stimulated overnight with BCG, and analyzed by multi-parameter flow cytometry to determine the frequency of cells producing either IFN-γ, TNF-α, or IL-2. The data are presented as the mean frequency ± SEM for 4 groups of mice. #, Significant differences between the cellular frequencies of the BCG vaccinated and the BCG vaccinated, malaria infected groups.
(TIF)
