# Supplementary figures and images for: Malaria Infections Do Not Compromise Vaccine-Induced Immunity against Tuberculosis in Mice
Source: PLoS One. 2011 Dec 19;6(12):e28164. doi: 10.1371/journal.pone.0028164 (PMC3242757; doi:10.1371/journal.pone.0028164)

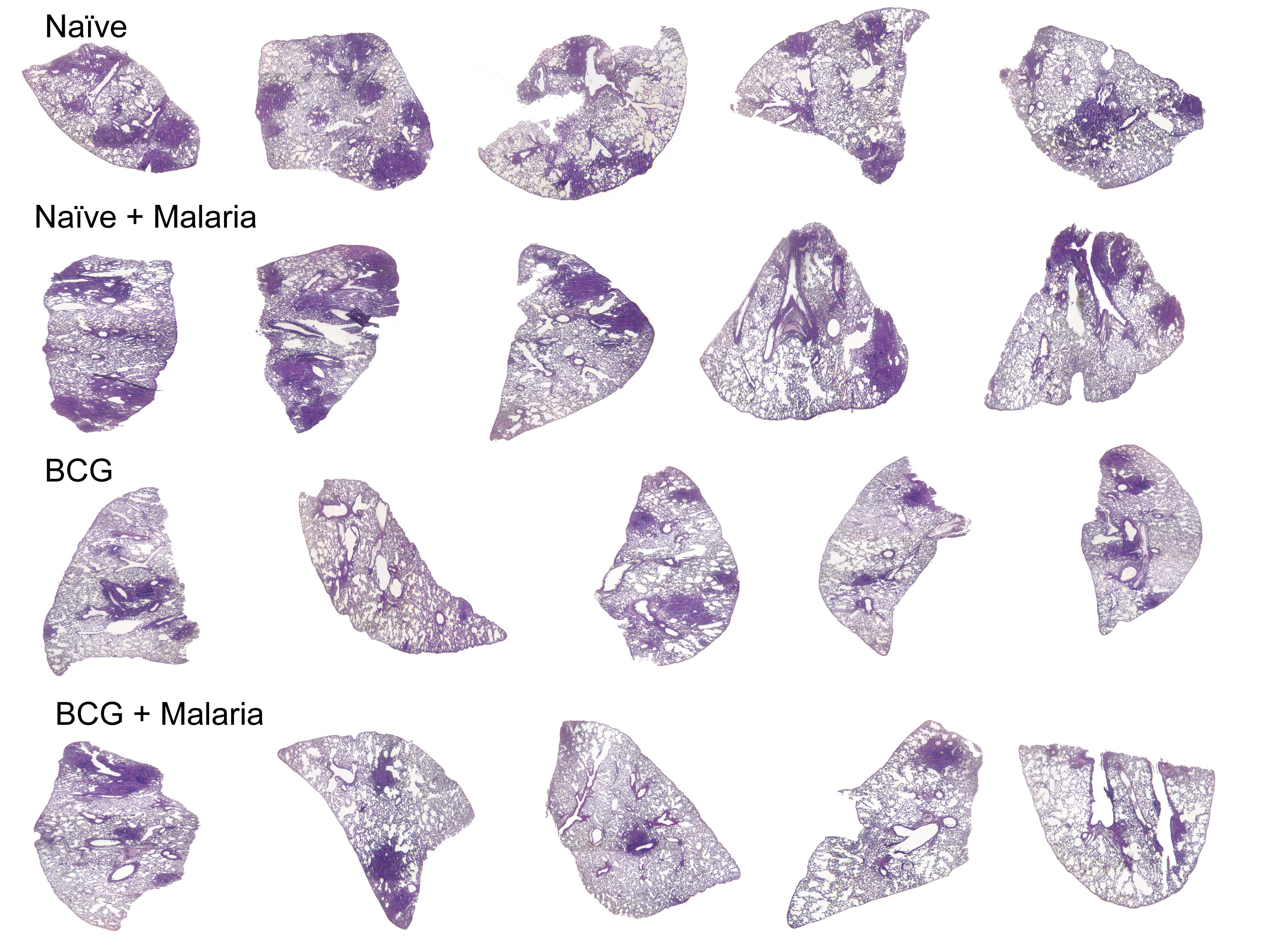

Supplement: Figure S1 — H & E stained lung sections from BCG vaccinated and malaria infected mice after a M. tuberculosis challenge by the aerosol route. Sections were obtained from naïve, BCG vaccinated, non-immunized-malaria infected and BCG vaccinated-malaria infected mice at 4 weeks after an aerogenic challenge with M. tuberculosis and analyzed by computer scanning using an Image pro analysis system. This analaysis showed no statistical diiferences in the inflammatory responses for BCG (20.2±6.4) and BCG/P. yoelii infected mice (20.9±9.32). Similarly, significant differences were not seen between the lung pathology values for naïve (38.6±10.6) and naïve/P. yoelii infected (34.9±3.9) animals. (TIF) [file pone.0028164.s001.tif]

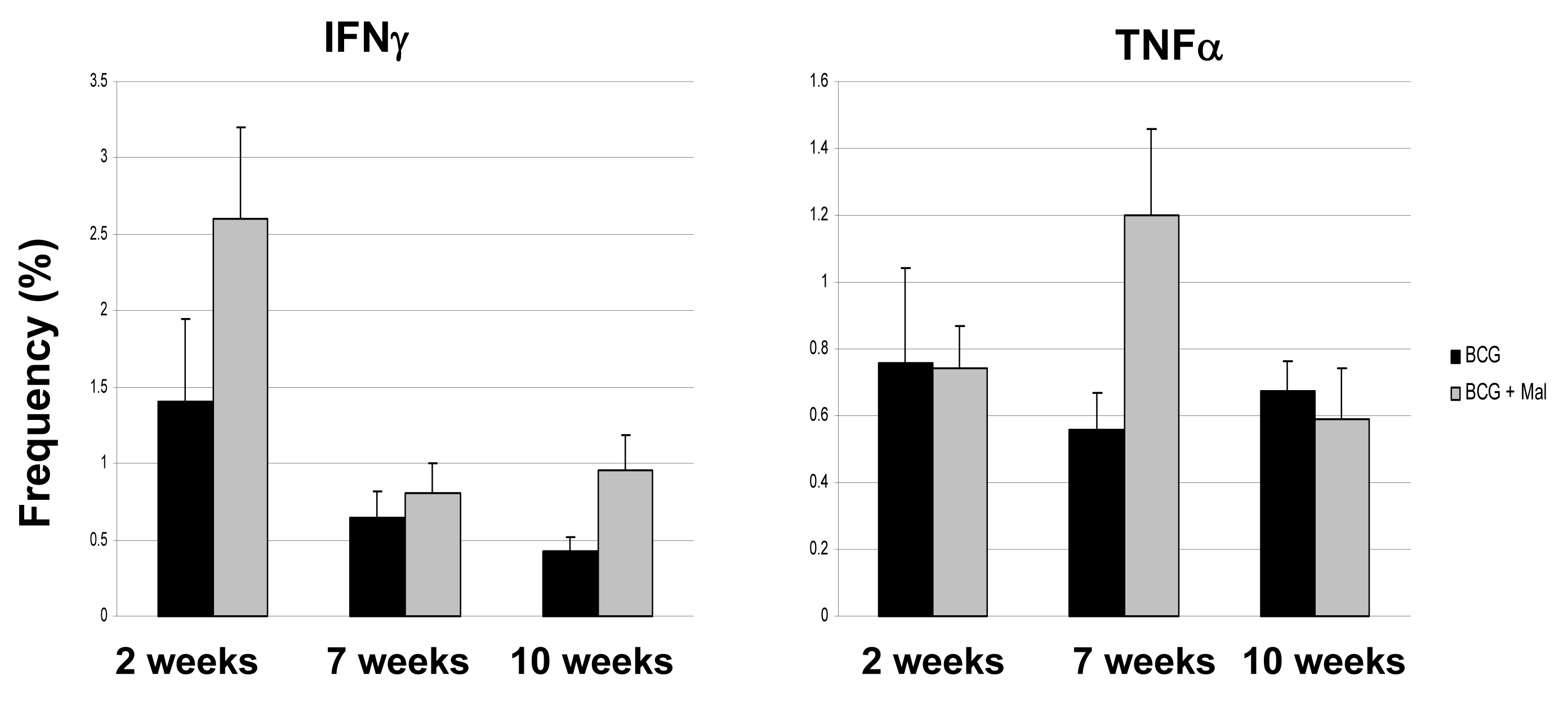

Supplement: Figure S2 — The frequency of CD4 (A) and CD8 (B) monofunctional cells recovered form the lungs of BCG vaccinated (black bars) and BCG vaccinated, malaria infected (grey bars) mice at 2, 7, and 10 weeks following the P. yoelii challenge. Lung cells were removed and pooled form 3 mice per group, stimulated overnight with BCG, and analyzed by multi-parameter flow cytometry to determine the frequency of cells producing either IFN-γ, TNF-α, or IL-2. The data are presented as the mean frequency ± SEM for 4 groups of mice. #, Significant differences between the cellular frequencies of the BCG vaccinated and the BCG vaccinated, malaria infected groups. (TIF) [file pone.0028164.s002.tif]
